# A Multiscale Recursive Attention Gate Federation Method for Multiple Working Conditions Fault Diagnosis

**DOI:** 10.3390/e25081165

**Published:** 2023-08-04

**Authors:** Zhiqiang Zhang, Funa Zhou, Chaoge Wang, Chenglin Wen, Xiong Hu, Tianzhen Wang

**Affiliations:** 1 School of Logistic Engineering, Shanghai Maritime University, Shanghai 201306, China; zqiang_zhang@foxmail.com (Z.Z.); cgwang@shmtu.edu.cn (C.W.); huxiong@shmtu.edu.cn (X.H.); tzwang@shmtu.edu.cn (T.W.); 2 Guangdong Provincial Key Lab of Robotics and Intelligent System, Shenzhen Institutes of Advanced Technology, Chinese Academy of Sciences, Shenzhen 518055, China; wencl@hdu.edu.cn; 3 School of AutoMation, Guangdong University of Petrochemical Technology, Maoming 525000, China

**Keywords:** federated learning, multiple working condition, multiscale recursive, fault diagnosis

## Abstract

Federated learning (FL) is an effective method when a single client cannot provide enough samples for multiple condition fault diagnosis of bearings since it can combine the information provided by multiple clients. However, some of the client’s working conditions are different; for example, different clients are in different stages of the whole life cycle, and different clients have different loads. At this point, the status of each client is not equal, and the traditional FL approach will lead to some clients’ useful information being ignored. The purpose of this paper is to investigate a multiscale recursive FL framework that makes the server more focused on the useful information provided by the clients to ensure the effectiveness of FL. The proposed FL method can build reliable multiple working condition fault diagnosis models due to the increased focus on useful information in the FL process and the full utilization of server information through local multiscale feature fusion. The validity of the proposed method was verified with the Case Western Reserve University benchmark dataset. With less local client training data and complex fault types, the proposed method improves the accuracy of fault diagnosis by 23.21% over the existing FL fault diagnosis.

## 1. Introduction

As a critical component of the motor, rolling bearings are susceptible to failure due to overload, aging, complex working conditions, and other factors [1,2,3]. When the data quality is better, the spectrum analysis can find the characteristic frequency of the fault and realize fault diagnosis. However, when the data quality is poor, the feature frequency of the fault is difficult to present on the spectrum [4,5].

As an effective data feature extraction tool, deep learning can extract features from raw data collected by sensors without relying on information about the remaining lifetime of critical components, and achieve end-to-end fault diagnosis by classifiers, and without the constraints of an exact physical model. Therefore, deep learning-based fault diagnosis research has received attention [6,7,8,9,10].

The amount of high-quality labeled data is a key factor limiting the effectiveness of deep learning fault diagnosis, but high-quality labeled data is difficult to obtain in the industry. When the operating load of the equipment changes, the statistical characteristics of the data collected by the monitoring sensors changes significantly, and this situation where the statistical characteristics change with the load is called multiple working conditions. The training samples collected from multiple working conditions violate the basic assumption of Independently Identically Distribution (i.i.d) required by the deep learning training mechanism, which affects the effectiveness of deep learning-based fault diagnosis and leads to unreliable fault diagnosis results. Therefore, it is necessary to carry out research on multiple working conditions fault diagnosis based on deep learning.

In fact, it is difficult to obtain multiple working condition samples, and the multiple working condition samples from a single client are not enough to build an accurate deep learning multiple working condition fault diagnosis model since the effectiveness of deep learning depends on the amount of data. On the other hand, the working conditions of the data may be different from one client to another, and the data of the client is usually confidential. Therefore, it is a worthwhile research problem to develop an effective multiple working condition fault diagnosis method jointly with multiple clients without sharing client data directly.

FL has received much attention from experts in the field of fault diagnosis in recent years due to its ability to accomplish collaborative training of multiple clients while ensuring data privacy. FL-based fault diagnosis aims to obtain a global model with satisfactory performance using information provided by multiple clients [11,12,13,14].

FL does initial feature mining on private data through local clients before sharing the mined information to the server for aggregation. The model parameters, gradients, and features of the client can be uploaded to the server as information for aggregation. Traditional FL-based fault diagnosis methods aggregate the information uploaded by the client to the server by averaging [15,16,17,18]. However, influenced by the different local data working conditions and model performance differences, the information uploaded by the clients contains both useful and useless information. The traditional FL method ignores the differences in the information provided by clients and assigns the same weight to different clients, which inevitably affects the fault diagnosis effect of all clients participating in the federation and makes the data of clients not fully utilized. Therefore, assigning different aggregation weights to different clients to make the server pay more attention to the useful information provided by the clients is a problem worth investigating.

**Remark** **1.** *Useful information from the client refers to information that contributes positively to the fault diagnosis results, and useless information refers to information that contributes negatively to the fault diagnosis results.*

This paper designs a multiscale recursive attention gate federation method for multiple working conditions fault diagnosis from the perspective of inter-client federation aggregation and local client feature extraction, including inter-client multiscale recursive federation strategy, and intra-client multiscale recursive fusion strategy. When the local model of the client receives new information, it continues training on the original model, which makes the feature representation of the neural network more powerful and thus makes the network output features more distinguishable. The purpose of optimizing local client feature extraction by using features from other clients is thus achieved. The method proposed in this paper can obtain a more powerful multiple working condition fault diagnosis model since useful information is better focused on both local client feature extraction and federal feature aggregation.

The contribution of this paper is as follows:

- A multiscale recursive attention-gate federation for multicondition fault diagnosis is designed, which can improve the accuracy of the inter-client federation and optimize the local multiple working conditions fault diagnosis effect by making full use of other client features.
- A multiscale recursive federation strategy for inter-client and a multiscale recursive fusion strategy for local clients are designed. A multiscale recursive federation policy located at the server can focus more on useful information uploaded by the client and improve the accuracy of the global features of the server. The locally located multiscale recursive fusion strategy can make the local client features and global features fully fused to achieve the purpose of optimizing the local multiple working condition fault diagnosis effect by making full use of the information from other clients.
- When there are differences in working conditions between data from different clients, using the method designed in this paper, satisfactory accuracy of multiple working conditions fault diagnosis can be achieved.

## 2. Related Work

Deep learning has received a lot of attention from experts in the field of fault diagnosis due to its ability to extract features from the raw signals monitored by sensors. Refs. [6,7] pointed out through their investigation that deep learning-based fault diagnosis does not depend on expert knowledge and accurate physical models and can achieve high-accuracy fault diagnosis. Wu et al. [8] designed a DNN-based adaptive bearing fault diagnosis architecture to enhance the generalization capability of the fault diagnosis model. Zhao et al. [9] designed a transfer learning approach for multiple working conditions fault diagnosis, which avoids the training of deep learning neural networks from scratch. Zhao et al. [10] designed a data-driven fault diagnosis method under data imbalance to improve the accuracy and robustness of the fault diagnosis model. However, the effectiveness of deep learning-based fault diagnosis depends on the number and quality of labeled samples, which is difficult to guarantee in a single client. Therefore, the research of fault diagnosis based on FL has been favored by experts.

**Figure 1:**
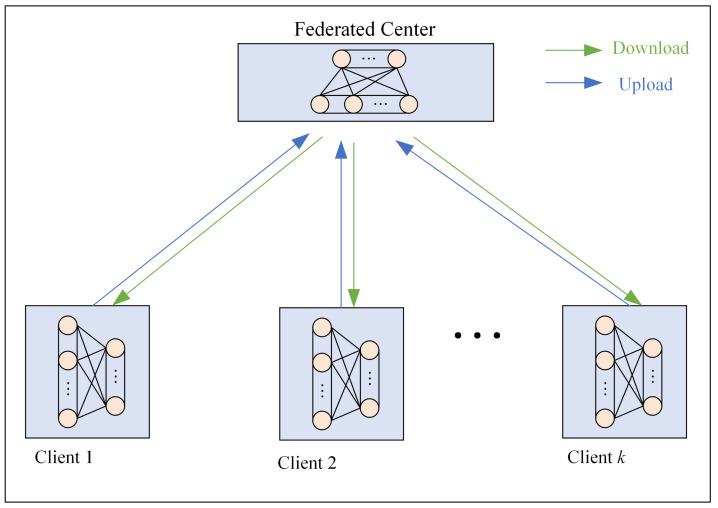
FL schematic.

FL is a distributed machine learning approach where clients use private data to train local models and then aggregate them at the model level without involving data sharing. In this way, FL combines the information of multiple clients without sharing data, which protects the privacy of local client data. The schematic diagram of FL is shown in Figure 1 [18,19]. FL-based fault diagnosis research aims to federate information from multiple clients to build global fault diagnosis models with powerful performance [20,21]. Throughout the parameter space of the neural network, the gradient descent method is used to find the optimal values of the parameters according to the direction of the gradient descent. Therefore, Wu et al. [22] used the gradient as information provided by the client to the server, which aggregated the information using averaging, and the aggregated information was fed back to the client for the next round of federation. The method realizes the joint training of multiple clients and improves the fault diagnosis accuracy of the global model. Zhang et al. [23] The model parameters of the client are uploaded to the server as client information for aggregation, which realizes the collaborative training of multiple clients and improves the fault diagnosis accuracy of the client. However, the above methods are affected by the accuracy of the local information on the client side. Therefore, some researchers are more concerned about whether the information uploaded by the client is useful or not. Zhang et al. [24] use the client’s local loss as the basis for judgment, and when the loss is greater than a specific threshold value, the client’s information is judged as useless and does not participate in the information aggregation of the round. This somewhat allows the server to aggregate more useful information, so the federation model outperforms traditional federation methods. Paragliola et al. [25] argue that as the number of layers of the network model deepens, the model information becomes more abstract, which is not conducive to the server focusing on the useful information of the model. Therefore, an efficient federation learning method is proposed, which sends not the complete model but a part of the client model from the local client to the federation center in each round of federation.

The above research method achieves collaborative optimization of multiple clients through different federation approaches, and the federation centers all use traditional federation averaging, giving the same weight to different clients. However, there are differences in the useful information provided by each client due to local data work and model performance, and Federated Averaging, (FedAvg) ignoring such differences will not fully exploit the advantages of FL. Therefore, this paper designs a multiscale recursive federation strategy among clients, which makes the server’s aggregation more focused on the useful information provided by the clients, and thus makes full use of the data from the clients. At the same time, the client local multiscale recursive fusion strategy is designed to fuse the global information into the local client feature extraction process, so that the local client features and global features are fully fused to achieve the purpose of using other client information to optimize the current client fault diagnosis effect.

## 3. Multiple Working Conditions Fault Diagnosis Method Based on Multiscale Recursive Attention Gate Federation

The purpose of FL-based fault diagnosis is to federate multiple clients to train a powerful global model, but the information uploaded to the server contains both useful and useless information when the local clients have different data conditions and model performance. The traditional FedAvg method ignores the differences in client information and assigns the same weight to different clients, which inevitably affects the fault diagnosis effect of all clients participating in the federation. Therefore, this section designs a multiscale recursive attention gate federation fault diagnosis method, which uses attention gates to give more attention to the useful information uploaded by the client, thus improving the accuracy of the client’s multiple working conditions fault diagnosis.

### 3.1. Multiscale Recursive Attention Gate Federation Method

Neural networks abstract features to higher scales through layer-by-layer feature representation, where features at different layers represent features at different scales. The feature representation of the same set of signals at different layers is different, and the distinguishability of features is also different.

On the other hand, it is difficult to obtain multiple working conditions data from local clients, so it is especially important to develop a multiple working conditions fault diagnosis method jointly with multiple clients. The simplest way to jointly develop multi-client fault diagnosis models is to share data, but sharing data involves the privacy information of clients. Therefore, the FL approach is that the clients locally do preliminary feature mining on private data first and then share the mined information, thus enabling multiple clients to jointly develop fault diagnosis models for multiple operating conditions. However, existing FL approaches do not take into account the differences in information uploaded by clients due to differences in local data and model performance. Therefore, a multiscale recursive attentional gate FL model (MAGFL) is designed in this section to improve the accuracy of multicondition fault diagnosis for the federation’s later client. The algorithm steps are as follows:

**Remark** **2.** *Shallow scale information is more comprehensive but coarser and less distinguishable. Deep-scale features are more accurate, but there is information loss. Therefore, the comprehensive use of multiscale features can improve the fault diagnosis accuracy of rolling bearings.*

**Step** **1:** **Designing a multiscale recursive FL framework between clients.**

**Figure 2:**
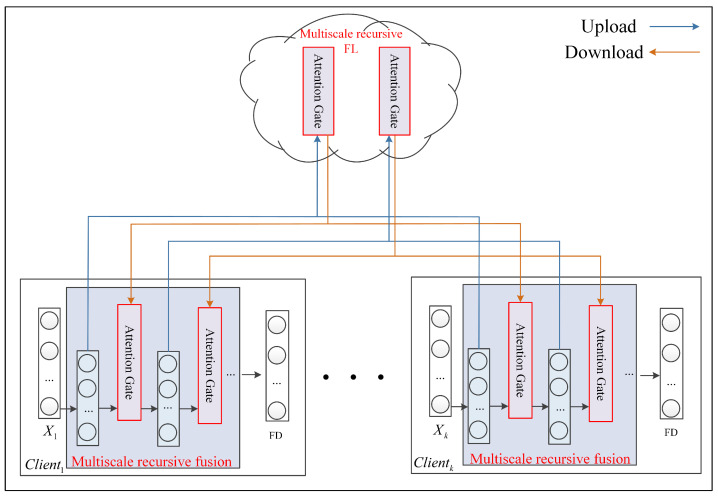
Multiscale recursive FL method.

The traditional FL method gives the same weight to the information uploaded by the client, which leads to the waste of useful information and thus affects the effectiveness of FL-based fault diagnosis. Therefore, a multiscale recursive FL framework among clients is designed as shown in Figure 2, which enables the FL model to focus more on the useful information provided by the clients.

For multiple clients $Client_{1},\ldots ,Client_{i},\ldots ,Client_{k}$, $X_{1}$ denotes the local data of $Client_{1}$, $X_{i}$ denotes the local data of $Client_{i}$, and $X_{k}$ denotes the local data of $Client_{k}$. In fact, each client may run in multiple working conditions, and each client may not run in exactly the same working condition. The server initializes the global model parameters $W_{global}^{0}=\left[\theta_{1},\ldots ,\theta_{j},\ldots ,\theta_{n}\right]$ and sends them down to the client, where θ denotes the network parameters of the model and *n* denotes the number of network layers. $\theta_{j}=\left[w_{j},b_{j}\right]$ denotes the weight $w_{j}$ and bias $b_{j}$ of the *j*-th layer. $Client_{i}$ starts local training using the model parameters inherited from the server as initial values.

$Client_{i}$ uses AutoEncoder (AE) for layer-by-layer feature extraction. Firstly, the first scale feature $h_{i,1}$ is extracted by the first AE, and then the first scale feature $h_{i,1}$ of each client is uploaded to the server for the first scale feature federation to obtain the server aggregated feature $F_{g,1}$ as shown in Equations (1) and (2).

(1) $$\begin{array}{cc} F_{g,1} & =AttGate(h_{1,1},\ldots ,h_{i,1},\ldots ,h_{k,1})\\ \; & =\left(1-Z_{1}\right)⊗h_{1}+Z_{1}⊗h_{1} \end{array}$$

(2) $$Z_{1}=\sigma \left(h_{1}W_{gate,s,1}+b_{gate,s,1}\right)$$

where AttGate(•) denotes the operator function of the attention gate, $h_{1}$ denotes the splicing feature of each client’s uploaded feature, splicing features means splicing of features by rows, from multiple features to one feature, $h_{1}=\left[h_{1,1},\ldots ,h_{i,1},\ldots ,h_{k,1}\right]$, and $W_{gate,s,1},b_{gate,s,1}$ denotes the aggregation parameters of the server’s attention gate. σ denotes the Sigmoid activation function of the neural network, and $Z_{1}$ is the weight assignment mechanism of the attention gate, obtained through a single layer of the neural network. ⊗ denotes the multiplication of the elements in the corresponding positions in the tensor. By using attention gates, it is possible to make the server’s aggregation more focused on the useful information in the features provided by each client, rather than giving equal weight to information from different clients. Attention gates achieve more attention to useful information by assigning different weights to the corresponding neurons. If the output of the attention gate is larger, it means that the corresponding neuron corresponding to the information is given a larger weight. The usefulness of the information depends on the contribution of the information output from the neuron to the fault diagnosis result, whether it is positive or negative.

Then, $F_{g,1}$ is distributed to each client for local multiscale feature fusion to obtain the client fusion feature $F_{f,i,1}$ as shown in Equation (3).

(3) $$F_{f,i,1}=MsFusion\left(h_{i,1},F_{g,1}\right)$$

where MsFusion(•) denotes the client’s local multiscale feature fusion strategy, which will be described in detail in step 2. Then, unsupervised feature extraction is performed on $F_{f,i,1}$ using AE to obtain the second-scale feature $h_{i,2}$. The $h_{i,2}$ is uploaded to the server for second-scale federal aggregation to obtain $F_{g,2}$ as shown in Equation (4).

(4) $$F_{g,2}=MsFed\left(h_{1,2},\ldots ,h_{i,2},\ldots ,h_{k,2}\right)$$

$F_{g,2}$ is then distributed to each client for local multiscale feature fusion. In such a way, the aggregated features $F_{g,n}$ at the *n*-th scale are obtained, and $F_{g,n}$ is sent down to the client for local multiscale feature fusion to obtain the fused features $F_{f,i,n}$, as shown in Equation (5).

(5) $$F_{f,i,n}=MsFusion\left(h_{i,n},F_{g,n}\right)$$

$F_{f,i,n}$ is then fed to the client’s Softmax classifier for local multiple working condition fault diagnosis.

**Step** **2:** **Multiscale recursive fusion within the client.**

**Figure 3:**
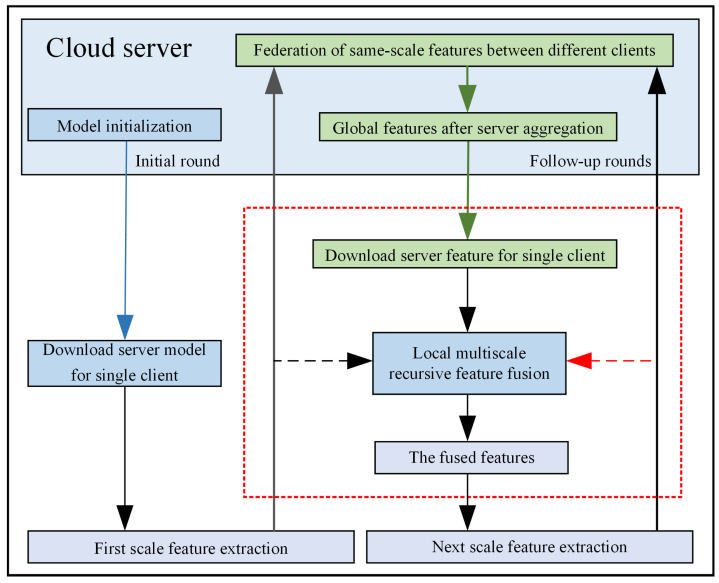
Information flow diagram of the proposed method.

The local layer-by-layer recursive use of global features provided by the server can make the global features better serve the current client. The information flow relationship of the proposed method is shown in Figure 3.

**Remark** **3.** *The features are essentially the outputs of the neurons in the hidden layer of the neural network. The mapping of information from data space to feature space is actually the transformation of the neural network from input to output. The reason is that the distinguishability of fault information in data space is not strong, and the purpose of transforming it to feature space by the neural network is to train the parameters of the neural network to make the distinguishability of fault information in feature space stronger.*

**Remark** **4.** *The client uses private data to train a local neural network model without requiring the sequential order of time series. In the process of FL, if the working conditions of each client are the same, then even the traditional FL method can obtain good fault diagnosis results. However, when it faces the problem of multiple working conditions, the fault diagnosis effect of the traditional FL method is not guaranteed. This paper focuses on fusing multiscale features of the client using a multiscale recursive federation approach, with attention gates used to focus on information useful to the client, thus solving the problem of multiple working conditions.*

For the *t*-th round of federation, the features $h_{i,1}$ at the first scale are first extracted locally at the client using AE, and then $h_{i,1}$ is uploaded to the server for aggregation to obtain $F_{g,1}$. The server sends $F_{g,1}$ down to the client locally for multiscale recursive fusion. $Client_{i}$ use the local multiscale recursive fusion of the inherited $F_{g,1}$ to obtain the fused features $F_{f,i,1}$. The fusion strategy uses the attention gate approach as shown in Equations (6) and (7).

(6) $$\begin{array}{cc} F_{f,i,1} & =AttGate(h_{i,1},F_{g,1})\\ \; & =\left(1-Z_{i,1}\right)⊗h_{i,1}+Z_{i,1}⊗F_{g,1} \end{array}$$

(7) $$Z_{i,1}=\sigma \left(h_{i,1}W_{gate,l,1}+b_{gate,l,1}\right)$$

By local multiscale recursive fusion of $F_{g,1}$ and $h_{i,1}$, the aggregated features can be better served for local client feature extraction, and the information from other clients can be used to optimize the effectiveness of multiple working conditions fault diagnosis for the current client.

Then, $h_{i,1}$ is mapped to a higher feature scale by AE to obtain $h_{i,2}$. The $h_{i,2}$ is uploaded to the server for federal aggregation at the second scale to obtain the aggregated feature $F_{g,2}$. $F_{g,2}$ contains useful information about all clients participating in the federation, which can be used to optimize the client’s local client feature extraction, so the client performs local recursive feature fusion of the inherited server features $F_{g,2}$ as shown in Equation (8).

(8) $$F_{f,i,3}=MsFusion\left(h_{i,3},F_{g,3}\right)$$

In such a way, the client locally performs n times of multiscale recursive fusion to obtain the top-level fused features $F_{f,i,n}$.

$F_{f,i,n}$ contains the features of all clients participating in the federation, and local multiscale recursive fusion can make the multiscale recursive federation work better so that the information from other clients can be used to improve the accuracy of local multiple working condition fault diagnosis.

In fact, different types of failures may occur when the client is working under different working conditions. The role of the attention gate is to selectively utilize the multiple working conditions information according to the local needs of the client. When different clients do not learn the same fault type information, the local client’s attention gate selects the information that is useful for their own fault diagnosis. The performance of the models differs from client to client when the length of the training set varies, which requires more attention to useful information, so attention gates are crucial to ensure the effectiveness of federal learning.

### 3.2. Multiple Working Condition Fault Diagnosis Based on MAGFL

**Figure 4:**
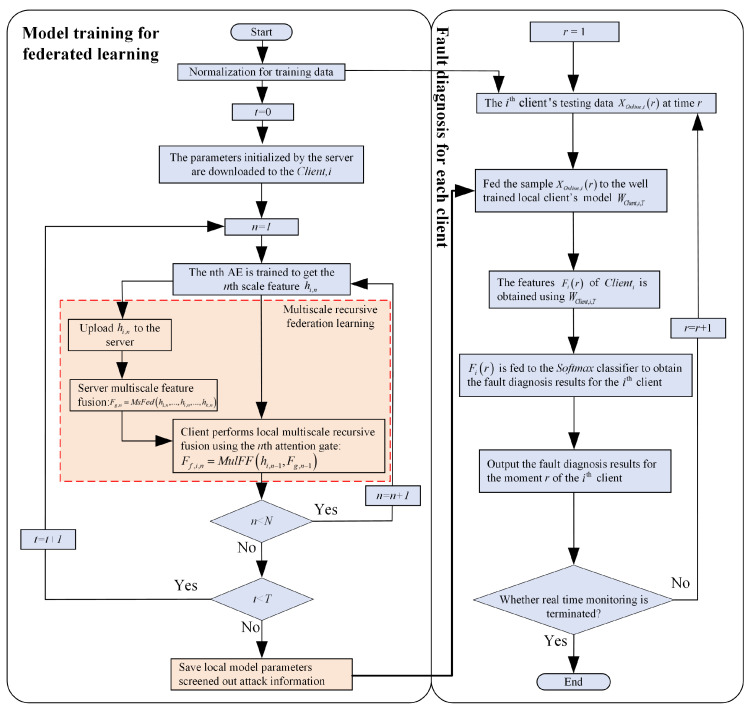
Flow chart of MAGFL-based fault diagnosis.

When the clients participating in the federation are running in different working conditions, they receive different useful information from the client to the server due to the difference in the quality of the client data. Traditional FedAvg methods ignore this difference in information and unselective averaging can lead to the propagation of useless information through the federation process, which does not guarantee the validity of the FL. The MAGFL-based fault diagnosis method is given in this section, and its flowchart is shown in Figure 4.

**Remark** **5.** *Traditional criteria for evaluating data quality include whether the data contains noise, whether there is an imbalance in the data, and whether the data is under network attack, etc. In deep learning, data quality is good if the data are conforming to independent homogeneous distribution. Because the data conform to independent homogeneous distribution is a prerequisite for the effectiveness of deep learning, and the effective features can be extracted by neural networks. If the data does not conform to the independent homogeneous distribution, then the quality of the data is poor.*

As shown in Figure 4, the fault diagnosis process based on the proposed method is divided into two parts: model training of multiscale recursive federation and client fault diagnosis of multiple working conditions. The left side of the figure indicates the model training part, and the red dashed box indicates the multiscale recursive federal process. When the training process is complete, each client saves their model parameters for fault diagnosis. Using the trained client parameters, features are extracted from the fault samples, and then the features are fed into the classifier to output the fault diagnosis results.

## 4. Experiment and Analysis

This section validates the effectiveness of the proposed method through experimental analysis using a benchmark dataset from Case Western Reserve University (CWRU) [26].

### 4.1. Description of Experimental Data

The CWRU vibration data used in this section is collected by an accelerometer mounted on the drive side of the motor with loads including 0, 1, 2, and 3 HP. The EDM technique was used to reintroduce single-point faults on the test bearings with fault sizes of 0.007, 0.014, and 0.021 inches. Vibration data are collected by an accelerometer, which is fixed to the drive and fan side of the motor housing using a magnetic base. The fault types and labels for the data used in this section are listed in Table 1. The 10 labels in Table 1 indicate the 10 types of faults.

### 4.2. Experimental Design

In order to verify the effectiveness of the proposed method, this section designs multiple working conditions fault diagnosis experiments, as listed in Table 2. Column 2 in Table 2 indicates the load of the device. 0/1/2/3 indicates that it contains four kinds of loads, which are 0 HP, 1 HP, 2 HP, and 3 HP. This section conducts experiments with different clients having different loads as an example to verify the effectiveness of the proposed method. Experiments 1–3 represent the experiments for fault diagnosis of 4 fault types with a fault size of 0.007 inches. Experiment 4–6 indicates the experiments of fault diagnosis for 10 fault types with fault sizes of 0.007, 0.014, and 0.021 inches. The models involved in the experimental comparison are listed in Table 3.

**Remark** **6.** *This paper starts the experimental verification with four clients, each containing one kind of load as an example. In fact, the equipment is in different stages of the life cycle, when the normal characteristics of the equipment will change, which is also a kind of multiple working conditions, and in addition, when the load or the working environment changes, the statistical distribution characteristics of the data will also change.*

This section uses a sliding window to process the raw vibration data to obtain the samples used in the experiment, with a window length of 900 and a sliding step size of 20. The sampling frequency of the data is 12 khz and the sampling period is 1/12,000 s. The length of the sliding window is 900 sample periods for 3/40 s. The sliding step is 20 sample periods for 1/600 s. If the sampling period is too small, the variability between two adjacent samples is small and can almost be regarded as the same sample, which contains less distinguishable information and will affect the results of fault diagnosis. This paper uses a DNN model with 3 hidden layers as the backbone model, the number of hidden layer neurons is 1200/1000/500, and the learning rate is 0.001. Local training is completed when the local training loss is less than the threshold, and the features are uploaded to the server with a loss threshold is 0.00001. The number of rounds in the federation is 100 rounds.

The running environment for the experiments is Python 3.7.11, Tensorflow 2.3.0 GPU. The computer is configured with an 11th generation Intel(R) Core(TM) i9-11900K 3.50 GHz, and it is manufactured by Intel Corporation, United States. and a NVIDIA GeForce RTX3090 GPU manufactured by NVIDIA in the United States. The operating system is Windows 10 Home.

### 4.3. Experimental Analysis

Different quality of data from clients provides different useful information to the server, and focusing on the useful information provided by clients will affect the effectiveness of federal learning fault diagnosis. The experimental results are listed in Table 4, Table 5, Table 6, Table 7, Table 8 and Table 9. Both Table 4 and Table 9 contain 7 rows and 6 columns, with the rows indicating the model and the columns indicating the fault diagnosis accuracy. This paper first designed experiment 1 for four types of faults with a fault size of 0.007 inches, and the experimental results are listed in Table 4.

Comparing the mean fault diagnosis accuracies of DNN and MCNN in Table 4, it can be seen that the accuracy of MCNN is 1.43% higher than that of DNN, which is due to the fact that MCNN uses different convolutional kernels to utilize the features of different scales of neural networks. Whereas DNN uses only the features of the last layer as input to the fault diagnosis classifier. A comparison of BNCNN and MCNN shows that the mean accuracy of multiple working condition fault diagnosis of BNCNN is 2.94% higher than that of MCNN, which is due to the fact that BNCNN uses BN to normalize the features after the convolutional layer, which is used to eliminate the effect of the difference in the distribution of multiple working condition data. Although the above-mentioned multiple working condition fault diagnosis methods do not need to preprocess the data in advance, the multiple working condition data from a single client is often insufficient to train an accurate deep learning fault diagnosis model. Therefore, joint training of multiple clients can be realized by FL.

Comparing FedAvg and BNCNN in Table 4, it can be seen that the mean fault diagnosis accuracy of FedAvg is 2.56% higher than that of BNCNN, which is due to the fact that FedAvg utilizes information from multiple clients in a comprehensive manner. Comparing the mean accuracy of DNN and FedAvg in Table 4, it can be seen that the average fault diagnosis accuracy of FedAvg is 6.93% higher than that of DNN due to the joint optimization between different clients implemented by FedAvg. However, the approach of treating all clients participating in the federation equally does not effectively utilize the information provided by the client. Comparing the mean accuracy of FedAvg and FedDv in Table 4, we can see that FedDv’s federation method obtains information from some clients for federation at a time, which reduces the interference of other clients and makes efficient use of the information provided by the clients, so FedDv’s diagnostic accuracy is 2.07% higher than FedAvg. However, FedDv still uses the average aggregation method, which causes the propagation of useless information. Comparing the mean accuracy of FedDv and FedLayer in Table 4, it can be seen that FedLayer has 2.12% higher accuracy than FedDv for multiple working condition fault diagnosis, and the utilization of the information provided by the client is improved due to the fact that a portion of the client information is aggregated each time.

However, the above approach does not take into account that the information provided by the client contains both useful and useless information, and the direct average aggregation approach does not take into account the propagation of useless information. At the same time, the client in the above method directly inherits the information of the global model as the initial value of the local optimization, without fully integrating the global information with the local information. The proposed method locally uses multiscale recursive feature fusion to utilize global information and fuses global features into the local client feature extraction process, which makes more full use of global information, so the multiscale recursive federation between clients and the local multiscale recursive feature fusion makes the proposed method achieve 99.09% fault diagnosis accuracy, and the fault diagnosis accuracy of each client is higher than existing methods.

Since the proposed approach allows the federation model to focus more on the useful information provided by the client, Experiment 2 and Experiment 3 were designed with a small amount of data from the client, and the results are listed in Table 5 and Table 6.

Comparing Table 4 and Table 5, it can be seen that the accuracy of multiple working condition fault diagnosis of each model decreases after the reduction in training data on the client side, which is due to the fact that less data means less useful information, so highlighting the use of useful information can improve the fault diagnosis accuracy. Comparing the mean accuracy of FedLayer and the proposed method in Table 5 shows that the proposed method is 15.41% higher than the existing method due to the highlighted utilization of useful information and also the local use of recursive fusion to optimize the local model using federal information.

**Figure 5:**
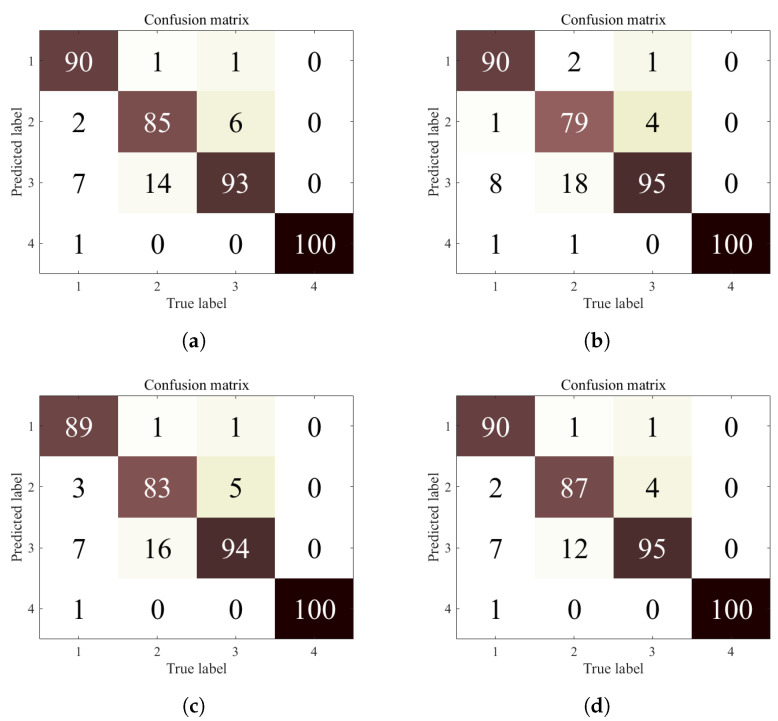
Confusion matrix of the proposed method in Experiment 3. (**a**) Confusion matrix of client 1. (**b**) Confusion matrix of client 2. (**c**) Confusion matrix of client 3. (**d**) Confusion matrix of client 4.

This helps us to conclude that the proposed approach is clearly superior to existing methods, especially when the available data on the client side is relatively small, as the utilization of useful information from limited data becomes particularly important at this point. In order to improve the readability of the experimental results, the confusion matrix of the experimental results of Experiment 3 is given in this section as shown in Figure 5. The rows of the confusion matrix in Figure 5 indicate the predicted label values of the model and the columns indicate the true label values of the samples. The diagonal lines indicate the number of samples with correct fault diagnosis results. As can be seen from Figure 5, the number of correct samples diagnosed by the proposed method is higher than all other methods, which reflects the superiority of the proposed method.

Comparing Table 5 and Table 6, it can be seen that the multiple working condition fault diagnosis accuracy of each model at a training sample size of 100 per class is lower than that at a training sample size of 500 per class. The difficulty of acquiring fault-labeled sample data in industrial sites has increased the need for an effective way of using the information to make full use of the useful information in existing data. The same conclusion can be drawn by comparing Table 4, Table 5 and Table 6. Therefore, in the fault diagnosis of multiple working conditions, making full use of the useful information provided by the clients participating in the federation and improving the effectiveness of the federation is an effective way to solve the difficulties in fault diagnosis of multiple working conditions. On the other hand, for the federal information inherited from the client, how to make it better optimize the local model is also an important means to improve the accuracy of client-side fault diagnosis.

To verify the effectiveness of the proposed model when the fault diagnosis task is more complex, experiments 4–6 were designed by increasing the fault types, and the fault diagnosis results when the fault types are ten classes and the number of training samples for each class is 1000 are listed in Table 7.

For a certain client, the complexity of the fault diagnosis task means that the types of faults to be diagnosed increase, but the number of samples in the training set remains the same, which will lead to a more difficult task of fault diagnosis. Therefore, comparing Table 4 and Table 7, it can be seen that although the number of training samples for each type of fault is 1000 for both clients, the more complex the fault diagnosis task is, the more difficult the diagnosis is, and the corresponding fault diagnosis accuracy is lower. In order to improve the fault diagnosis accuracy for multiple working conditions, it is necessary to improve the effectiveness of the federation on the one hand, and to make full use of the global information provided by the server on the other hand locally. When performing the fault diagnosis task with 10 classifications, the fault diagnosis accuracy of each model after reducing the number of training samples to 500 for each class is listed in Table 8.

**Figure 6:**
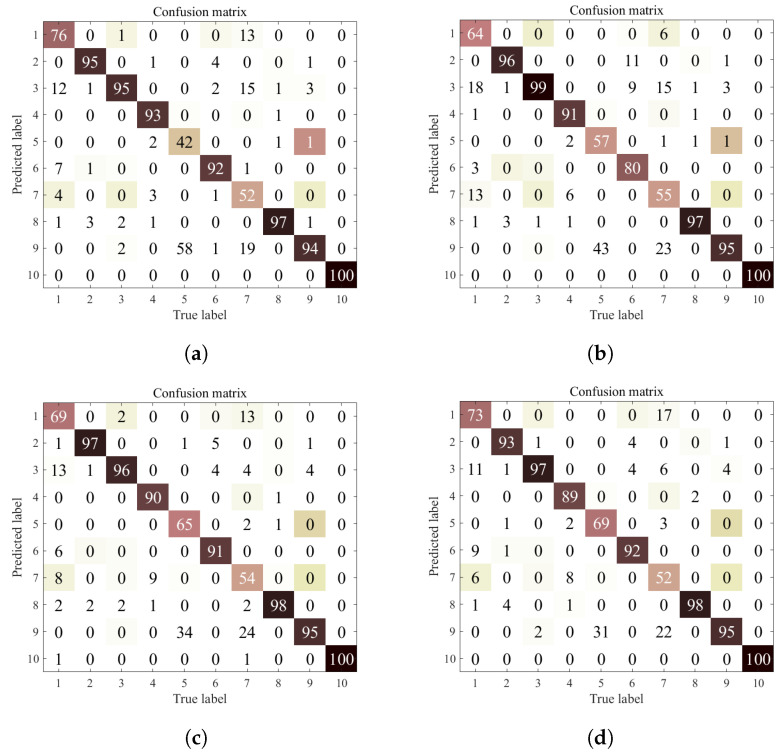
Confusion matrix of the proposed method in Experiment 6. (**a**) Confusion matrix of client 1. (**b**) Confusion matrix of client 2. (**c**) Confusion matrix of client 3. (**d**) Confusion matrix of client 4.

Comparing Table 7 and Table 8, it can be seen that decreasing the number of training samples with the same number of fault types, the fault diagnosis accuracy of each model decreases, indicating that the useful information in the training samples decreases and obtaining usable fault information from a small number of samples becomes a key factor for accurate fault diagnosis. Comparing Table 5 and Table 8, we can see that although the number of training samples for each category is 500, the more types of faults need to be diagnosed, the more difficult the fault diagnosis is, so the fault diagnosis accuracy of ten categories is lower than that of four categories. However, the degradation of fault diagnosis accuracy is minimal for the method proposed in this paper, which indicates that the method proposed in this paper has stronger information extraction ability. To further test the effectiveness of the proposed method, the number of training samples was continued to be reduced to 100 per class, and the experimental results are listed in Table 9. To improve the readability of the experimental results, a confusion matrix of the experimental results is given in this section as shown in Figure 6. The rows of the confusion matrix in Figure 6 indicate the predicted label values of the model and the columns indicate the true label values of the samples. The diagonal lines indicate the number of samples with correct fault diagnosis results.

**Figure 7:**
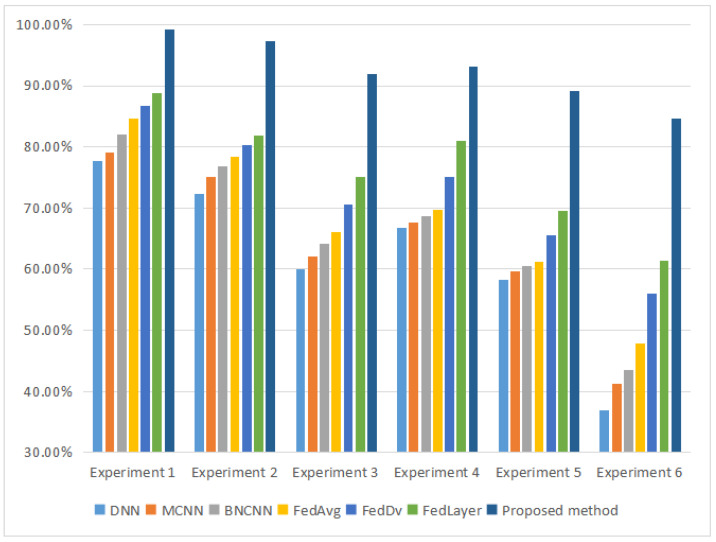
Histogram of experimental results.

Comparing Table 7, Table 8 and Table 9, it can be seen that the existing FL fault diagnosis method can accomplish effective federated learning fault diagnosis jointly with multiple clients under the premise that the clients have sufficient training samples. However, it is difficult to label the data in industrial sites, and the data with fault labels are often less, so the method proposed in this paper is more superior when there are more fault types and less training sample data. The histogram of the experimental results is shown in Figure 7. The Y-axis in the figure indicates the fault diagnosis accuracy.

This paper takes Experiment 1 as an example to analyze the variance and standard deviation of the fault diagnosis accuracy of each model. Each model performs fault diagnosis 20 times, and the variance and standard deviation of the fault diagnosis results are calculated from the fault diagnosis results 20 times. The experimental results are shown in Table 10.

From Table 10, it can be seen that the standard deviation of the fault diagnosis results of MCNN and BNCNN is less than that of DNN due to the use of multiscale features. The standard deviation of the algorithms for fault diagnosis through federated learning is smaller than that of the method without federation, indicating that FL is more robust. The proposed method has the smallest standard deviation of 0.0084 for the fault diagnosis results due to the multiscale recursive federation, indicating the good robustness of the proposed method.

## 5. Conclusions and Future Work

The purpose of FL is to accomplish joint optimization of multiple clients without sharing data directly, and the information uploaded to the server by each client is given the same aggregated weight. However, the information uploaded to the server by each client is not equal due to the change in working conditions, and directly assigning equal weights to all clients’ information will ignore the attention to useful information and affect the effect of federal learning fault diagnosis for multiple working conditions. Therefore, the multiscale recursive FL framework aims to make the server pay more attention to the useful information provided by the model and ignore the useless information through attention gates to ensure that the information provided by the client is fully utilized during each federation. On the other hand, for the global information aggregated by the server, the client uses the designed multiscale recursive fusion, which can effectively fuse the global information into the local client feature extraction process and achieve the effect of using other client information to optimize the current client information. The designed multiscale recursive federation among clients and the multiscale recursive fusion method locally by clients can complete the joint optimization of each client in the case of obvious differences in client work conditions, and make full use of the useful information provided by clients. The experimental results show that the proposed method is 23.21% higher than the existing FL fault diagnosis method when the client faces a complex multiple working condition fault diagnosis task and the available training data is relatively small. The experimental results show that the use of the proposed method can give more attention to the useful information provided by the client in the process of federation.

This study used a small amount of labeled data from a single customer and did not consider the large amount of unlabeled data collected during the operation of the device. Therefore, future work should design a semi-supervised federal learning mechanism for the case of small amounts of labeled data.

## Figures and Tables

**Table 1:** Details of experimental data.

Fault Type	Fault Size (inch)	Label
Ball fault	0.007	1
Inner race fault	0.007	2
Outer race fault	0.007	3
Ball fault	0.014	4
Inner race fault	0.014	5
Outer race fault	0.014	6
Ball fault	0.021	7
Inner race fault	0.021	8
Outer race fault	0.021	9
Normal	0	10

**Table 2:** Experimental design.

Experiment	Load of Client (HP)	Number of Training Set Samples	Number of Test Set Samples
Experiment 1	0/1/2/3	4 × 1000	4 × 100
Experiment 2	0/1/2/3	4 × 500	4 × 100
Experiment 3	0/1/2/3	4 × 100	4 × 100
Experiment 4	0/1/2/3	10 × 1000	10 × 100
Experiment 5	0/1/2/3	10 × 500	10 × 100
Experiment 6	0/1/2/3	10 × 100	10 × 100

**Table 3:** Relevant experimental models.

Model	Model Explanation
DNN	DNN without federation.
MCNN [9]	A method for multiple working condition fault diagnosis using multiple convolutional kernels to obtain multiscale features.
BNCNN [10]	Adding batch normalization (BN) after the convolutional layer is used to eliminate the distributional differences in multiple cases.
FedAvg [21]	Aggregate the information provided by the client using averaging.
FedDv [24]	A portion of the clients’ information is participating in the federation instead of all of them.
FedLayer [25]	A part of the client’s information is involved in the federation and not all of it.
Proposed method	Multiscale recursive attention-gate federation method with local use of multiscale recursive fusion.

**Table 4:** Fault diagnosis accuracy for four types of faults with a sample size of 1000 for each type of fault.

	Client 1	Client 2	Client 3	Client 4	Mean
DNN	79.00%	78.75%	78.75%	74.00%	77.63%
MCNN	81.25%	79.75%	80.25%	75.00%	79.06%
BNCNN	83.00%	82.25%	83.00%	79.75%	82.00%
FedAvg	84.25%	84.25%	84.75%	85.00%	84.56%
FedDv	86.75%	86.25%	86.25%	87.25%	86.63%
FedLayer	88.00%	89.00%	88.50%	89.50%	88.75%
Proposed method	99.00%	99.00%	99.25%	99.25%	99.13%

**Table 5:** Fault diagnosis accuracy for four types of faults with a sample size of 500 for each type of fault.

	Client 1	Client 2	Client 3	Client 4	Mean
DNN	72.25%	71.00%	72.50%	73.50%	72.31%
MCNN	75.00%	75.75%	74.25%	75.25%	75.06%
BNCNN	76.25%	77.50%	76.75%	77.00%	76.88%
FedAvg	78.75%	78.75%	77.00%	78.75%	78.31%
FedDv	80.25%	80.50%	79.25%	81.25%	80.31%
FedLayer	82.75%	81.25%	81.25%	82.00%	81.81%
Proposed method	96.88%	96.75%	97.50%	97.76%	97.22%

**Table 6:** Fault diagnosis accuracy for four types of faults with a sample size of 100 for each type of fault.

	Client 1	Client 2	Client 3	Client 4	Mean
DNN	57.25%	61.00%	60.00%	61.75%	60.00%
MCNN	60.25%	62.00%	62.75%	63.50%	62.13%
BNCNN	63.75%	64.25%	64.00%	64.75%	64.19%
FedAvg	66.50%	65.25%	66.00%	66.50%	66.06%
FedDv	70.00%	71.75%	70.25%	70.50%	70.63%
FedLayer	74.50%	75.75%	75.25%	75.00%	75.13%
Proposed method	92.00%	91.00%	91.50%	93.00%	91.88%

**Table 7:** Fault diagnosis accuracy for ten types of faults with a sample size of 1000 for each type of fault.

	Client 1	Client 2	Client 3	Client 4	Mean
DNN	66.56%	67.62%	66.27%	66.83%	66.82%
MCNN	67.46%	68.04%	67.75%	67.24%	67.62%
BNCNN	68.56%	68.41%	68.63%	69.23%	68.71%
FedAvg	70.04%	69.62%	69.32%	70.15%	69.78%
FedDv	74.57%	74.96%	75.73%	74.86%	75.03%
FedLayer	81.30%	80.41%	81.62%	80.68%	81.00%
Proposed method	92.25%	93.60%	93.00%	93.43%	93.07%

**Table 8:** Fault diagnosis accuracy for ten types of faults with a sample size of 500 for each type of fault.

	Client 1	Client 2	Client 3	Client 4	Mean
DNN	56.55%	59.13%	58.12%	59.10%	58.23%
MCNN	58.36%	60.38%	59.53%	60.28%	59.64%
BNCNN	59.57%	60.85%	60.47%	60.95%	60.46%
FedAvg	61.32%	61.54%	61.21%	60.45%	61.13%
FedDv	65.63%	65.35%	65.74%	65.38%	65.53%
FedLayer	69.73%	69.27%	69.93%	69.02%	69.49%
Proposed method	88.90%	88.89%	89.50%	89.40%	89.17%

**Table 9:** Fault diagnosis accuracy for ten types of faults with a sample size of 100 for each type of fault.

	Client 1	Client 2	Client 3	Client 4	Mean
DNN	35.19%	37.55%	37.90%	36.72%	36.84%
MCNN	40.35%	41.39%	42.85%	40.37%	41.24%
BNCNN	42.78%	43.20%	43.63%	44.63%	43.56%
FedAvg	47.43%	48.50%	48.04%	47.47%	47.86%
FedDv	55.57%	56.24%	55.82%	56.35%	56.00%
FedLayer	60.39%	61.46%	62.19%	61.45%	61.37%
Proposed method	83.60%	83.40%	85.50%	85.80%	84.58%

**Table 10:** Variance and standard deviation of model fault diagnosis results.

	DNN	MCNN	BNCNN	FedAvg	FedDv	FedLayer	Proposed Method
Variance	1.6 × 10${}^{-3}$	1.3 × 10${}^{-3}$	6.46 × 10${}^{-3}$	1.83 × 10${}^{-4}$	1.96 × 10${}^{-4}$	1.346 × 10${}^{-4}$	7.1 × 10${}^{-5}$
Standard Deviation	0.0396	0.0362	0.0254	0.0135	0.014	0.0116	0.0084

## Data Availability

The data involved in this article have been presented in the article.
